# Optimization of the production and characterization of milk clotting enzymes by *Bacillus subtilis* natto

**DOI:** 10.1186/2193-1801-2-33

**Published:** 2013-01-31

**Authors:** Fang-Chen Wu, Chen-Wei Chang, Ing-Lung Shih

**Affiliations:** 1 Department of Bioindustry Technology, Da-Yeh University, Changhua, Taiwan; 2 Department of Environmental Engineering, Da-Yeh University, 168, University Rd, Dacun, Changhua 51591 Taiwan

**Keywords:** *Bacillus subtilis* natto, Milk-clotting enzyme, Milk-clotting activity, Cheese making, Ultrafiltration, Response surface methodology

## Abstract

Suitable medium for production of milk clotting enzyme (MCE) by *Bacillus subtilis* (natto) Takahashi in submerged liquid-state fermentation was screened, the nutrient factors affecting MCE production was optimized by response surface methodology. The MCE production by *B*. *subtilis* (natto) Takahashi was increased significantly by 428% in the optimal medium developed. The MCE was filtered and concentrated by ultrafiltration. The retentate after tandem filtration carried out with the combined membranes of MWCO 50kDa and 5 kDa showed two major bands between 25kDa and 30kDa on SDS-PAGE, and the MCA and MCA/PA improved significantly in comparison with those in the initial broth. The crude enzyme thus obtained showed MCA and MCA/PA ratio of 48,000 SU/g and 6,400, which are commensurate with those (MCA 26,667 SU/g and MCA/PA 6,667) of the commercial rennet. It had optimal pH and temperature at pH 6 and 60°C, and showed excellent pH and thermal stability.

## Introduction

Milk coagulation is a basic step in cheese manufacturing. For a long time calf rennet, the conventional milk clotting enzyme obtained from the fourth stomach of suckling calves (Nagodawithana & Reed 1993 ), is the most widely used coagulant in cheese-making all over the world to manufacture most of the cheese varieties. The worldwide reduced supply of calf rennet and the ever increase of cheese production and consumption have stimulated the research for milk clotting enzyme (MCE) from alternative sources to be used as calf rennet substitutes (Areces et al. 1992 ; Escobar & Barnett 1993 ; Lopes et al. 1998 ; Nouani et al. 2011 ). Various animals, plants and microbial proteases have been suggested as milk coagulants (Chazarra et al. 2007 ; D’Ambrosio et al. 2003 ; Silva & Malcata 2005 ; Zhang et al. 2011 ). However, attention has been focused on the production of milk-clotting enzymes (MCEs) from microbial sources for use as rennin substitutes (Ayhan et al. 2001 ; Cavalcanti et al. 2004 ; Hashem 1999 ; Silveira et al. 2005 ). Although there are many microorganisms that produce MCEs (Ding et al. 2011 ; He et al. 2011 ; Li et al. 2012 ; Vishwanatha et al. 2010 ), only the MCEs produced by strains of *Rhizomucor miehei*, *Rhizomucor pusillus* var. *Lindt*, *Aspergillus oryzae* and *Enthothia parasitica* are widely used (Birkkjaer & Jonk 1985 ; Crawford 1985 ; Thakur et al. 1990a ).

*Bacillus subtilis* (natto) Takahashi, a commercial natto starter, is commonly used to prepare fermented soybean product-natto, which is a traditional Japanese food for more than 1,000 years. *Bacillus subtilis* is one of the most investigated microbial groups, because they can produce varieties of biotechnological interesting substances (Schallmey et al. 2004 ; Shih & Yu 2005 ); it is known to secrete several proteases during the fermentation process (Rao et al. 1998 ). The capacity of selected *Bacillus* strains to produce and secrete large quantities of extracellular enzymes has led them to be among the most important industrial enzyme producers. While *B*. *subtilis* (natto) produces many enzymes, including amylases and cellulases, the most important enzymes in the production of natto are proteases; many of them have been characterized (Yoshimoto et al. 1971 ). The proteases are responsible for the main flavor, through hydrolysis of soybean protein. It is conceivable that *B*. *subtilis* (natto) may produce MCE(s). Recently, we have shown that *Bacillus subtilis* (natto) Takahashi produced milk clotting enzyme (MCE). The Milk-clotting activity (MCA) and milk-clotting activity/proteolytic activity (MCA/PA) ratio of the crude enzyme obtained are comparable with those of Pfizer microbial rennin and *Mucor* rennin (Shieh et al. 2009 ).

Previous works on the optimization of parameters for MCE production were conducted using " one-factor-at -a-time" technique (Ayhan et al. 2001 ; Cavalcanti et al. 2004 ; Hashem 1999 ; Silveira et al. 2005 ). Unfortunately, it frequently fails to locate the region of optimum response because the joint effects of factors on the response are not taken into account in such procedure. It was reported that the complexities and uncertainties associated the large-scale microbial fermentation usually come from lack of knowledge of the sophisticated interactions among various factors. The response surface methodology (RSM) has been increasingly used for various phases of an optimization process in fermentation (Shih & Shen 2006 ; Shih et al. 2002 ). It is a powerful technique for testing multiple process variables because fewer experimental trials are needed compared to the study of one variable at a time. In addition, interactions between variables can be identified and quantified by such technique (Box & Wilson 1951 ). However, the application of this technique on optimizing the MCE production was scarce in the literature. In the present study, we screened suitable medium for production of MCE and applied RSM to optimize the medium content for the enhancement of MCE production by *Bacillus subtilis* (natto) Takahashi in submerged liquid-state fermentation (SLF) and characterized the MCE thus produced.

## Material and methods

### Bacteria strains and reagents

*B*. *subtilis* (natto) Takahashi was obtained from Takahashi Yuzo research facility Japan. Reagents for cultivation such as nutrient agar (NA), nutrient broth (NB) were purchased from DIFCO Laboratories Michigan, USA. Potassium dihydrogenphosphate (NaH_2_PO_4_), sodium nitrate (NaNO_3_), ammonium chloride (NH_4_Cl), ferrous Sulfate (FeSO_4_ 7H_2_O) and starch were purchased from Katayama Chemical Inc. (Osaka, Japan). Corn steep powder (CSP), MgSO_4_⋅7H_2_O, NaH_2_PO_4_⋅2H_2_O, Na_2_HPO_4_ 12H_2_O, rennin from *Rhizomucor miehei* (type II) were obtained from Sigma Chemical (St. Louis, MO). Dry skim milk powder was from New Zealand milk brands Ltd. All other reagents used were of the highest grade available unless indicated otherwise.

### Fermentation media and conditions

#### Preparation of inoculums

*B*. *subtilis* (natto) Takahashi or other tested *Bacillus subtilis* (natto) bacteria were first cultured on NA (Difco Laboratories) containing agar (15 g l^-1^), beef extract (3 g l^-1^), peptone (5 g l^-1^) at 37°C overnight. The colonies appeared on the plate were picked up (1 cm square) and inoculated into 5 ml of NB composed of beef extract (3g l^-1^), peptone(5 g l^-1^) in a 30 ml test tube. The medium was adjusted to pH 7.0 and then incubated at 37°C, for 20 h with shaking at 175 rpm. After incubation, 1ml of bacteria were inoculated into 100 ml medium composed of beef extract (3g l^-1^), peptone (5 g l^-1^), soybean (50g l^-1^), sucrose (2 g l^-1^) and NaCl (5 g l^-1^) in a 250 ml Erlenmyer flask. The culture broth was adjusted to pH 7.0, incubated at 37°C for 20 h with shaking at 175 rpm, which was then used as inoculums for the later experiments.

#### Liquid state fermentation (LSF)

In the preliminary experiments, six media were screened for their suitability for MCE production; they included the liquid fermentation medium (designated as LF medium in this paper) used in the previous report (Shieh et al. 2009 ) and five other different media commonly used by researchers investigating production of MCE by *Rhizomucor miehei* (Thakur et al. 1990b ). LF medium was composed of sucrose (50 g l^-1^), NaCl (10 g l^-1^), MgSO_4_.7H_2_O (0.5 g l^-1^), NaH_2_PO_4_.2H_2_O (3 g l^-1^) and NaHPO_4_.12H_2_O (3 g l^-1^). The other five media were designated as M1, M2, M3, M4 and M5. M1was composed of starch (30 g l^-1^), corn steep powder (5 g l^-1^), soybean meal (2 g l^-1^), dry milk (12 g l^-1^), KH_2_PO_4_ (2 g l^-1^), NaNO_3_ (1 g l^-1^), NHCl_4_ (1 g l^-1^) and FeSO_4_.7H_2_O (0.01 g l^-1^). M2was composed of starch (4 g l^-1^), soybean meal (5 g l^-1^), CaCO_3_ (5 g l^-1^) and ground barley (10 g l^-1^). M3 was composed of glucose (10 g l^-1^), soybean meal (30 g l^-1^), dry milk (10 g l^-1^), KH_2_PO_4_ (0.05 g l^-1^), NaNO_3_ (10 g l^-1^), MgSO_4_.7H_2_O (0.025 g l^-1^). M4 was composed of rice bran (100 g l^-1^), soybean meal (10 g l^-1^), rice flour (10 g l^-1^), CaCl_2_.7H_2_O (10 g l^-1^). M5 was composed of starch (100 g l^-1^), lactose (4.3 g l^-1^), soybean meal (30 g l^-1^), yeast extract (3 g l^-1^), MgSO_4_.7H_2_O (0.025 g l^-1^). The culture media were steamed and sterilized in autoclave at 121°C for 15 min. Each medium was inoculated with 5ml (5%, v/v) of bacteria inoculums prepared above and adjusted to pH 7.0, followed by incubation at 37°C with shaking at 175 rpm for 72 h. At the end of incubation, the culture medium was filtered through the cotton cloth to remove insoluble, which was followed by centrifugation at 5,600 x g, 4°C for 20 min to remove the bacteria and the supernatant was then filtered through a 0.45μm filter. The liquid broth of the crude enzyme was used to assay for milk-clotting activity. To investigate the factors affecting MCE production, the cultivation was carried out using the medium and conditions indicated above except that the factors were varied by experimental design described below.

#### Assay for milk clotting activity

Milk clotting activity was determined according to the method of Arima (Arima et al. 1970 ) and expressed in terms of Soxhlet units (SU). One SU is defined as the amount of enzyme which clots 1ml of a solution containing 0.1 g skim milk powder and 0.00111 g calcium chlorides in 40 min at 35°C. In brief, 0.5 ml of tested materials was added to a test-tube containing 5ml of reconstituted skim milk solution (10g dry skim milk/100ml, 0.01 M CaCl_2_) pre-incubated at 35°C for 5 min. The mixture was mixed well and the clotting time T (sec), the time period starting from the addition of test material to the first appearance of clots of milk solution, was recorded and the clotting activity was calculated using the following formula: SU= 2400x5xD/Tx0.5; T= clotting time (sec); D=Dilution of test material. The test materials include liquid solution of crude enzyme from LSF and commercial rennet of *Rhizomucor miehei*.

#### Assay of protease activity

The proteolytic activity was determined at pH 6.5 by the casein digestion method ( Kunitz 1947 ) and expressed as optical density (OD) at 660 nm. Ratio of the milk-clotting activity to proteolytic activity is expressed as milk-clotting units (SU) versus the OD_660_’s obtained in the proteolytic measurements.

#### Protein determination

Protein determination was done according to the method of Lowry et al (Lowry et al. 1951 ).

#### RSM Experimental design

In preliminary studies ( Chang 2010 ; Shieh et al. 2009 ), we used "one-factor-at-a-time" technique to evaluate various factors for their suitability to sustain good production of MCE by *B*. *subtilis* (natto) Takahashi. The preliminary data suggested that starch (X_1_), corn steep powder (X_2_), soybean meal (X_3_) and dry milk (X_4_) were the major variables ( Chang 2010 ). As is seen below (Results and discussions), the preliminary result shows that MCA was 600 SU/ml when *B*. *subtilis* (natto) Takahashi was incubated in M1 in which starch, corn steep powder, soybean meal and dry milk was 30 g l^-1^, 5 g l^-1^, 2 g l^-1^ and 12 g l^-1^, respectively. Therefore, these four factors were chosen for further optimization through RSM.

#### Factorial design

In the first experiment of this series, the ranges of the variables tested were starch (30 g l^-1^), corn steep powder (5 g l^-1^), soybean meal (2 g l^-1^) and dry milk (12 g l^-1^). For a 2^4^ factorial design with four factors at two levels, sixteen experimental runs are required (Box & Wilson 1951 ). Two center points were added to estimate the experimental error and check the adequacy of the first-order model. Although two-level (full or fractional) factorial experiments will only yield data to fit a limited model (equation 1 or 2), they are the most common initial experiments in the application of RSM, because orthogonality of the design minimizes the variance of the regression coefficients (Maddox & Richert 1977 ; Montgomery 1991 ). Table 1 shows the four independent variables and their concentrations at the different coded levels of the factorial design experiments. The matrix corresponding to the 2^4^ factorial designs, together with the observed experimental data and predicted values from model equation are also shown in Table 1. To avoid bias, the total of 18 runs was performed in a random order (overall randomization).
12

**Table 1:** **Experimental design and results of the four**-**factor**-**two**-**level factorial design together with the predicted yields from the pure first**-**order model**

Factors^a^	X_1_(g/L)	X_2_(g/L)	X_3_(g/L)	X_4_(g/L)	Observed MCA(SU/mL)	Predicted MCA(SU/mL)
Trial no.						
1	10	2	1	8	28.57	84.1162
2	50	2	1	8	533.33	639.2012
3	10	8	1	8	12.31	−31.3188
4	50	8	1	8	505.26	523.7662
5	10	2	3	8	40	86.6212
6	50	2	3	8	685.71	641.7063
7	10	8	3	8	25.8	−28.8138
8	50	8	3	8	417.39	526.2712
9	10	2	1	16	64	142.4388
10	50	2	1	16	738.46	697.5237
11	10	8	1	16	25	27.0037
12	50	8	1	16	564.71	582.0888
13	10	2	3	16	53.33	144.9438
14	50	2	3	16	800	700.0288
15	10	8	3	16	12.31	29.5088
16	50	8	3	16	457.14	584.5938
^b^17(C)	30	5	2	12	533.33	334.355
^b^18(C)	30	5	2	12	521.74	334.355

^a^ Starch (X_1_), Corn steep powder (X_2_), Soybean meal (X_3_) and Dry milk (X_4_); ^b^ Centrepoints.

Y is the predicted response (MCA in this study, SU/ml); β_0_, β_i_, β_ij_ are constant coefficients, and x_i_, x_j_ are the coded independent variables or factors.

#### Central composite design (CCD)

Based on the results obtained from the factorial design, Box–Wilson central composite design (CCD) was used to optimize the levels of variables, which can help to identify and quantify the interaction between variables (Box & Wilson 1951 ). In addition, to fully explore the subregion of the response surface in the neighborhood of the optimum, an experimental design with more than two levels of each factor is required, so that a second order approximation to the response surface can be developed; a CCD with five coded levels was used for this purpose. The four factors are starch (X_1_), corn steep powder (X_2_), soybean meal (X_3_) and dry milk (X_4_) and the levels of the variables for the CCD experiments were chosen in reconciliation with the data of our previous experiments. For the four factors, this trial was essentially a full 2^4^ factorial design augmented by eight axial points (or called star points) coded ±α and three replications of center point (all factors at level 0), resulting in a total number of 27 experiments (Box & Wilson 1951 ). The distance of the star points from the centrepoint is given by α=2^n/4^ (for four factors n=4, α=2.0). For predicting the optimal point, the experimental data of a CCD are usually used to fit a second-order polynomial equation (equation 3), as it gives a complete picture including any possible interaction between the values. The matrix corresponding to the CCD is shown in Table 2, together with the observed experimental data and predicted values from the model equation.
3

Y is the predicted response (MCA in this study, SU/ml); β_0_, β_i_, β_ii,_

β_ij_ are constant coefficients, and x_i_, x_j_ are the coded independent variables or factors.

**Table 2:** **Experimental design and results of the central composite design together with predicted yields from the model equation**

Factors^a^	X_1_(g/L)	X_2_(g/L)	X_3_(g/L)	X_4_(g/L)	MCA(SU/mL)	Predicted Values
Trial						
1	20	3	1.5	8	40.00	99.639
2	40	3	1.5	8	600.00	600.917
3	20	7	1.5	8	20.00	85.639
4	40	7	1.5	8	533.33	534.747
5	20	3	2.5	8	133.33	231.47
6	40	3	2.5	8	800.00	783.084
7	20	7	2.5	8	80.00	120.305
8	40	7	2.5	8	600.00	619.749
9	20	3	1.5	16	233.33	297.304
10	40	3	1.5	16	800.00	826.417
11	20	7	1.5	16	25.00	108.639
12	40	7	1.5	16	600.00	585.582
13	20	3	2.5	16	333.33	398.635
14	40	3	2.5	16	960.00	978.084
15	20	7	2.5	16	30.00	112.805
16	40	7	2.5	16	633.33	640.084
17	10	5	2	12	30.00	−174.501
18	50	5	2	12	800.00	854.056
19	30	1	2	12	700.00	617.442
20	30	9	2	12	333.33	265.442
21	30	5	1	12	450.00	381.611
22	30	5	3	12	650.00	567.944
23	30	5	2	4	450.00	390.777
24	30	5	2	20	700.00	608.777
^b^25(C)	30	5	2	12	533.33	542.167
^b^26(C)	30	5	2	12	571.43	542.167
^b^27(C)	30	5	2	12	521.74	542.167

^a^Starch (X_1_), Corn steep powder (X_2_), Soybean meal (X_3_) and Dry milk (X_4_) ; ^b^Centrepoints.

#### Software for experimental design and statistical analysis

Statistica, version 6.0 (Statsoft, Inc., Tulsa, OK USA) was used for the experimental design and regression analysis of the experimental data obtained. The quality of fit of the model equation was expressed by the coefficient of determination R^2^, and its statistical significance was determined by an F-test. The significance of the regression coefficients was tested by a t-test. For analysis of the nature of the fitted response and for prediction of the maximum point, the second-order equation was reduced to its canonical form (Maddox & Richert 1977 ; Montgomery 1991 ). Canonical analysis was one part of the Statistica output.

#### Concentration of MCE by ultrafiltration

The cell-free supernatant of the culture was cycled though a Tami ultrafiltration membrane system equipped with various ceramic membranes (the molecular weight cut-off (MWCO); 1kDa, 5 kDa, 50 kDa, Quebec, Canada). The apparatus was operated with a transmembrane pressure (TMP) of 100 psi, and room temperatures between 20 and 35°C. During the separation, the product is fractionated in two phases: the concentrated retentate and the filtrate. Both concentrated retentate and the filtrate were used to assay for milk-clotting activity as described above, and their protein contents were determined according to the method of Lowry (Lowry et al. 1951 ). The molecular weight of MCE was determined by sodium dodecyl sulfate-polyacryamide gel electrophoresis (SDS-PAGE) according to the method of Weber and Osborn (Weber & Osborn 1969 ).

#### pH and thermal stability

The thermal stability was determined by pre-incubating the enzyme in the temperature range of 40-80°C. The incubation time of samples varied from 5 to 120 min. After incubation, the samples were submitted for determination of residual milk-clotting activity. To study the pH stability of MCE produced by *B*. *subtilis* (natto) Takahashi, the enzyme was held at 35°C, different pH levels (pH 5-10) for up to 72 h, followed by activity assay as described above. The buffers used were according to the method described previously (Vishwanatha et al. 2010 ).

## Results and discussion

### *Screening of suitable medium for MCE production in LSF by B*. *subtilis* (natto) Takahashi

**Figure 1:**
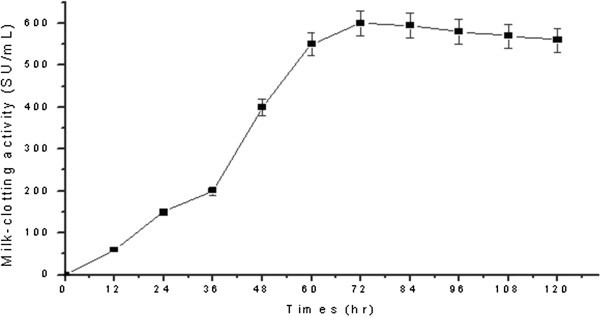
**Time course of milk**-**clotting activities produced by*Bacillus subtilis*** (**natto**) **Takahashi in medium M1.**

Previously, it was shown that *B*. *subtilis* (natto) Takahashi produced MCE in LF medium, the milk-clotting activity (MCA) and milk-clotting activity/proteolytic activity (MCA/PA) ratio of the crude enzyme obtained were comparable with those of Pfizer microbial rennin and *Rhizomucor* rennin (Shieh et al. 2009 ). Therefore, it is a strain that showed potential for further study to produce industrial useful MCE as a calf rennet substitutes. M1, M2, M3, M4 and M5 indicated above were the media of choice for researchers investigating production of MCE by *Rhizomucor miehei* (Thakur et al. 1990b ). They were tested for the suitability of MCE production by *B*. *subtilis* (natto) Takahashi, and the results are shown in Table 3. The highest MCA and MCA/PA ratio (600 SU/ml and 4,878) reached was for the cultivation in M1, which is inconsistent with the results shown for *Rhizomucor miehei* in that the highest MCA (1,482 SU/ml) reached was for its cultivation in M2 (Thakur et al. 1990b ). In addition, the highest MCA and MCA/PA ratio (600 SU/ml and 4,878) reached when *B*. *subtilis* (natto) Takahashi was cultured in M1 were comparable and improved in comparison with those (685 SU/ml and 2,981) obtained when it was cultured in LF medium previously used (Shieh et al. 2009 ). As shown in Figure 1, when the *B*. *subtilis* (natto) Takahashi were cultivated in M1, the MCA increased dramatically after 36 h of cultivation to reach a maximum value at 72 h of cultivation and then declined, this is consistent with the results obtained for *Rhizomucor miehei* cultivated in M1, M2, M3 and M4 medium. To further optimize the MCE production by *B*. *subtilis* (natto) Takahashi in M1 medium, the effects of some important nutrient parameters on the productions of MCE were investigated by RSM.

**Table 3:** **Comparison of MCE produced by*B***. ***subtilis*** (**natto**) **Takahashi in various medium with its commercial counterparts**

Medium^a^	Milk-clotting activity ()	Protease activity (OD_660_)	Ratio ()
M1	600 ± 12	0.123 ± 0.012	4878 ± 476
M2	26.67 ± 2.10	0.050 ± 0.006	533 ± 64
M3	(t>40min)	0.043 ± 0.011	NA
M4	160 ± 10	0.076 ± 0.015	2105 ± 451
M5	80 ± 4	0.027 ± 0.008	2963 ± 877
LF	685 ± 14	0.230 ± 0.028	2981 ± 367
**Commercial Protease**			
Mucor rennin	511 ± 13	0.11 ± 0.02	4650 ± 845
P- Rennin^b,c^	750	0.29	2590
Papain^c^	216	0.59	367
Pepsin^c^	2	0.015	133

^a^Cultivated in shake flask at 37 °C, 175 rpm, pH 7.0 for 72h; ^b^Microbial rennin from *Endothia parasitica*.; ^c^Published results from Arima, 1970 (Arima et al. 1970 ).

### *Optimization of MCE production by B*. *subtilis* (natto) Takahashi in M1 medium *using RSM*

Application of RSM requires the identification of the major factors that are suitable to sustain good production of MCE. In the preliminary studies of MCE production by *B*. *subtilis* (natto) Takahashi in LF and M1 medium ( Chang 2010 ; Shieh et al. 2009 ), it appeared that *B*. *subtilis* (natto) Takahashi would produce milk-clotting enzyme under the condition of limited organic nitrogen supply and oversupply of inorganic nitrogen sources inhibited the production of milk-clotting activities by this bacterium, a phenomenon that was sustained by the similar observation in the MCE production by a variety of other microorganisms such as *Rhizomucor miehei* (Thakur et al. 1990a ), *Streptomyces clavuligerus* (Porto et al. 1996 ), *Aspergillus versicolor* (Abdel-Fattah & Saleh 1979 ) and *Mucor baciliformis* (Areces et al. 1992 ). In addition, it was also found that carbon sources were critical for MCE production by *B*. *subtilis* (natto) Takahashi. However, the requirement of carbon source for MCE production varied depending on the microorganism used (Abdel-Fattah & Saleh 1979 ; Cavalcanti et al. 2005 ; Hashem 1999 ; Shieh et al. 2009 ). Therefore, starch (X_1_), corn steep powder (X_2_), soybean meal (X_3_) and dry milk (X_4_) were the major variables in M1 affecting the performance of the culture in terms of MCE production, and they were chosen for optimization by RSM; the inorganic nitrogen sources were omitted for further study. Initially, a complete four-factor-two-level factorial design was carried out, which was followed by the central composite design (CCD) to optimize the factors for MCE production.

### Results of factorial experiments

The experimental results of MCE productions are shown in Table 1. In order to approach the vicinity of the optimum, a first-order model was fitted to the data obtained from the factorial design experiment. From the analysis of the data in Table 1 by the least-squares method, the regression coefficients and corresponding t values for the model were obtained. Accordingly, the fitted model with coded variables is shown in equation 4.

Pure First-order Model4

According to the analyses of variances (ANOVA), F value for the overall regression is significant at 5% level and the lack of fit is insignificant indicating that the pure first-order model is very adequate in approximating the response surface of the experimental design. This statement is further supported by the satisfactory value of the coefficient of determination R^2^ (0.8945). Judging from the regression coefficients and the corresponding t values, it is concluded that the linear terms of starch, corn steep powder and dry milk had significant effect on MCE production; it is predicted that increasing the concentration of starch (X_1_), dry milk (X_4_) and decreasing the concentration of corn steep powder (X_2_) should enhance MCE production. In contrast, the soybean meal (X_3_) at the tested range exhibited insignificant effect on MCE production.

#### Results of CCD experiments

By applying multiple regression analysis on the experimental data shown in Table 2, the experimental results of the CCD were fitted with the polynomial equation 3, and the second-order polynomial equation obtained is shown in equation 5.

Second-order Model Equation5

Judging from the regression coefficients and corresponding t values, it was concluded that the linear term of starch (X_1_), corn steep powder (X_2_), soybean meal (X_3_) and dry milk (X_4_) and the cross term of starch (X_1_) displayed significant effect on MCE production at a 5% level (p<0.05). However, all the other terms were not significant at a 5% level. The fit of the model was checked by the coefficient of determination R^2^, which was calculated to be 0.9416, indicating that 94.16% of the variability in the response could be explained by the model. According to the analysis of variance (ANOVA), the test statistics F values for the overall regression is significant at the upper 5% level, which further supported that the second-order model is very adequate in approximating the response surface of the experimental design. For the analysis of the fitted surface, equation 5 was transformed into its canonical form (equation 6).6

The coefficients of the equation 6 are eigenvalues based on coded data, and Y is the MCA (SU/ml). Since all coefficients of the above equation are all negative, the response surface is suggested to have a maximum point (1048.02 SU/ml) where the optimum combination of concentration is starch 55.41 g l^-1^, corn steep powder 1.5 g l^-1^, soybean meal 2.69 g l^-1^ and dry milk 22.29 g l^-1^, respectively. Verification of the calculated maximum was done with experiments that were performed in the culture media representing the optimum combination found, and the MCE production of 1043.48 SU/ml (average of three repeats) was obtained. The excellent correlation between predicted and experimental values justifies the validity of the response model.

### Concentration of MCE by ultrafiltration and its characterization

**Figure 2:**
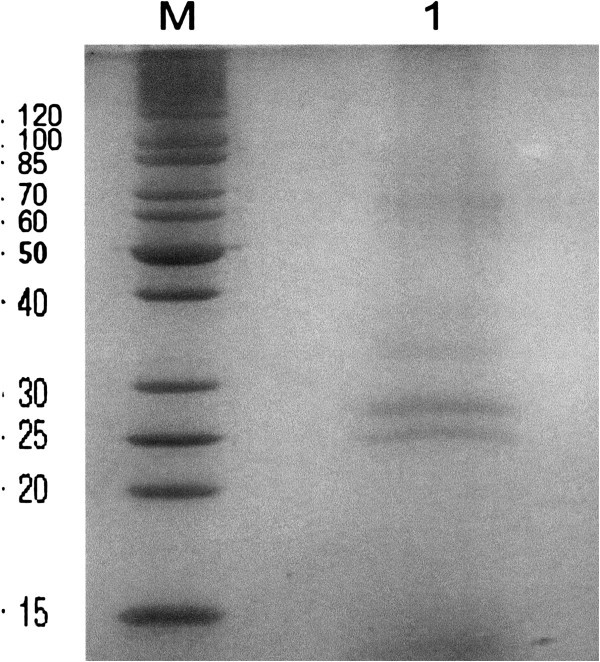
**SDS**-**PAGE of the crude enzyme obtained after tandem filtration using combined membranes of 50kDa and 5 kDa.** Line M: Marker; Line 1: crude enzyme.

Properties of various samples from fractionation by ultrafiltration, which was cycling cell-free supernatant of the culture through MWCO of 5 kDa and 50 kDa membranes, are shown in Table 4. The data showed that after filtering through MWCO of 5 kDa membrane, the MCA and MCA/PA of the retentate was 3.33-fold and 3.54-fold of those in the initial broth supernatant; the MCA and MCA/PA of the filtrate however was 0.24-fold and 0.31-fold of those in the initial broth supernatant, indicating that the majority of MCE was retained within the 5 kDa membrane. In contrast, after filtering through MWCO of 50 kDa membrane, the MCA and MCA/PA of the retentate was 0.5-fold and 0.55-fold of those in the initial broth supernatant; the MCA and MCA/PA of the filtrate however was 1.25-fold and 1.30-fold of those in the initial broth supernatant, indicating that most of MCE was filtered through the 50 kDa membrane. When tandem filtration was carried out with the combined membranes of 50kDa and 5 kDa, the SDS-PAGE of the retentate showed two major bands between 25kDa and 30kDa (Figure 2), which is comparable with many MCE reported in the literature; Shindo and Arima (Shindo & Arima 1979 ) reported molecular weight of calf chymosin to be 35 kDa by SDS-PADE. Similarly, molecular weight has been reported to be 36 kDa and 37.5 kDa for lamb chymosin (Baudys et al. 1988 ; Rogelj et al. 2001 ). In addition, it was reported that purified MCE of *Bacillus sphaericus* appeared as two bands having molecular mass of 25 and 47 kDa on SDS-PAGE (ElBendary et al. 2007 ); the molecular weight of partially purified MCE of *Amylomyces rouxii* is about 47.5 kDa infered from SDS-PAGE and Native-PAGE, 48.6 kDa inferred from gel filtration (Yu & Chou 2005 ).

**Table 4:** **Properties of various samples from fractionation by ultrafiltration**

Activity	MCA (SU/mL)	Protein Con. (mg/mL)	Proteolytic activity (OD660)	MCA/PA Ratiio
Fraction				
Initial Broth	960 ± 9	0.188 ± 0.006	0.115 ± 0.003	8348 ± 231
Concentrated Retentate	3200 ± 80^a^ (480 ± 12)^b^	0.231 ± 0.005 ^a^ (0.174 ± 0.008)^b^	0.108 ± 0.004 ^a^ (0.104 ± 0.003)^b^	29630 ± 1323^a^ (4615 ± 176)^b^
Filtrate	228 ± 11^a^ (1200 ± 25)^b^	0.116 ± 0.010^a^ (0.224 ± 0.009)^b^	0.089 ± 0.004^a^ (0.111 ± 0.005)^b^	2561 ± 168^a^ (10811 ± 535)^b^

^a^Values calculated for samples obtained using membrane having MWCO 5 kDa.

^b^Values (in the parenthesis) calculated for samples obtained using membrane having MWCO 50 kDa.

The retentate after tandem filtration carried out with the combined membranes of 50kDa and 5 kDa was further lyophilized, the crude MCE powder obtained was re-dissolved and tested for the enzyme activity. The MCA and MCA/PA ratio of the crude enzyme thus obtained was 48,000 SU/g and 6,400 (data not shown), which are commensurate with those (MCA 26,667 SU/g and MCA/PA 6,667) of the commercial rennet from Sigma.

### pH and thermal stability

**Figure 3:**
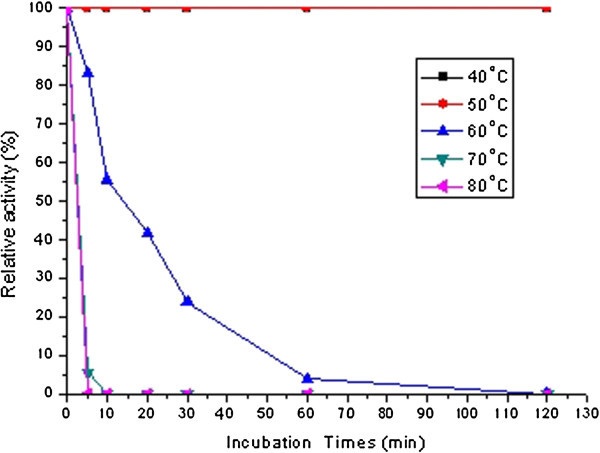
**Thermal**-**stability of milk**-**clotting enzyme produced by*Bacillus subtilis*** (**natto**) **Takahashi.**

**Figure 4:**
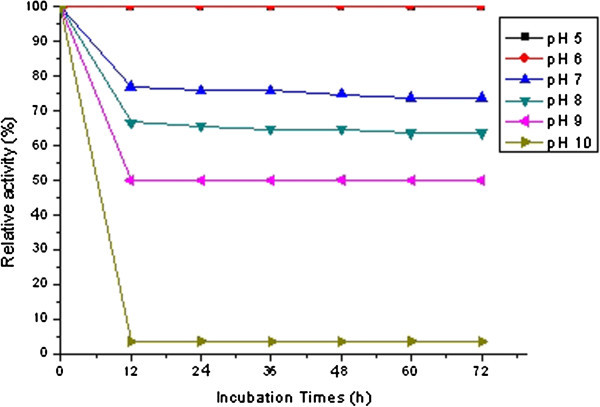
**pH**-**stability of milk**-**clotting enzyme produced by*Bacillus subtilis*** (**natto**) **Takahashi.**

The enzyme obtained above had optimal pH and temperature at pH 6 and 60°C (data not shown), and it showed excellent thermal and pH stability (Figures 3 and 4). The enzyme completely retained its MCA even after incubation for more than 2 h at pH 6, and temperature at 40°C and 50°C (Figure 3). However, the MCA decreased dramatically when temperature increased; the enzyme lost 50% of MCA after it was incubated at 60°C for 20 min and it was deactivated completely at temperature more than 70°C within 10 min of incubation. The MCE by *B*. *subtilis* (natto) Takahashi shown in this study sustained higher thermal-stability than those by various microorganisms. The MCE(s) by *P*. *oxalicum* ( Hashem 2000 ), *M*. *pusillus* (Khan et al. 1979 ) and *Mucor* J20 (Tubesha & Al- Delaimy 2003 ) were completely inactivated upon heating at temperature higher than 55°C in less than 20 min.

The results shown in Figure 4 indicated that the enzyme completely retained its activity at pH 5 and pH 6 for more than 72 h. However, the MCA decreased while the pH increased; after 12 h of incubation, the enzyme retained 77%, 68% and 50% of its activity at pH 7, pH 8 and pH 9, respectively. The MCA was completely diminished after incubation for 12 h at pH 10. The optimum pH for the MCE of *B*. *subtilis* (natto) Takahashi is different from that of the partially purified enzyme of other microorganism; it is pH 4, pH 5.5 and pH 11 for the MCE of *P*. *oxalicum* ( Hashem 2000 ), *M*. *baciliformis* (Venera et al. 1997 ) and *Nocardiopsis* sp. (Cavalcanti et al. 2004 ) respectively. However, the pH stabilities for the MCE(s) of these microorganisms were rarely reported and obscure. The fact that the MCE of *B*. *subtilis* (natto) Takahashi showed a wide rang of pH stability, in that it retained more than 50% of its activity between pH 5-9 for more than 72 h at 35°C, has made it more useful as a cheese-making coagulant because pH-sensitive coagulants generally lead to reduced yields and defective cheese (Cavalcanti et al. 2004 ).

## Conclusions

The MCE production by the *B*. *subtilis* (natto) Takahashi was increased significantly by 428%, from 600 SU/ml in M1, a medium conventionally used in the literature, to 1048.02 SU/ml in the optimal medium developed by surface response methodology (RSM), indicating that RSM is proven to be a powerful and useful tool for optimizing the substrate concentration for enhancing MCE production. The MCE was successfully concentrated by application of ultrafiltration. The crude enzyme obtained after ultrafiltration showed two major bands between 25kDa and 30kDa on SDS-PAGE, which is consistent with many other MCE reported. The crude enzyme thus obtained showed MCA and MCA/PA ratio of 48,000 SU/g and 6,400, which are commensurate with those (MCA 26,667 SU/g and MCA/PA 6,667) of the commercial rennet. In addition, it had optimal pH and temperature at pH 6 and 60°C, and showed excellent pH and thermal stability. The data shown have suggested that *B*. *subtilis* (natto) Takahashi is an ideal strain for the production of the MCE that has potential as calf rennet substitutes and it is a good choice for further studies and industrial exploitations.
